# Gender-related beliefs and attitudes about tobacco use and smoking cessation in Mexico

**DOI:** 10.1080/21642850.2021.1935963

**Published:** 2021-06-10

**Authors:** Rosibel Rodríguez-Bolaños, Marta Caballero, Guadalupe Ponciano-Rodríguez, Luz María González-Robledo, Francisco Cartujano-Barrera, Luz Myriam Reynales-Shigematsu, Ana Paula Cupertino

**Affiliations:** a Population Health Research Center, Mexican National Institute of Public Health, Morelos, Mexico; b Faculty of Higher Studies of Cuautla, Universidad Autónoma del Estado de Morelos, Morelos, México; c Public Health Department, Faculty of Medicine, National Autonomous University of Mexico, Ciudad de México, Mexico; d School of Medicine, Universidad Autónoma del Estado de Morelos, Cuernavaca Morelos, Mexico; e James P. Wilmot Cancer Institute, University of Rochester Medical Center, Rochester, NY, USA

**Keywords:** Gender, smoking cessation, primary health care, sex differences, Mexico

## Abstract

Background. While overall trends in tobacco use among men are declining, tobacco use continues to rise significantly among women in developing countries. This study aimed to explore the gender-related beliefs and attitudes about tobacco use and smoking cessation in Mexico, one of the top five Latin America countries with the highest prevalence of tobacco consumption.

Materials and Methods. This study was conducted using an explanatory qualitative methods design. Semi-structured interviews were conducted with 14 adults smokers (8 women & 6 men) who visited primary healthcare clinics in Mexico City. Two researchers independently coded the interviews and applied the final codes upon consensus. Inter-rater reliability was assessed for four groups of codewords (92% agreement), based on an ecological model on socio-cultural factors.

Findings. Initiation to smoking in women begins out of curiosity, and in men by imitation. Also, women start using tobacco at an older age compared to men. During maintenance of smoking, women report experiencing loneliness and anxiety about multiple responsibilities, e.g. women reveal that they feel guilty when they smoke due to their maternal role as caregivers. Additionally, some women report that smoking is a symbol of freedom, recalling the media messages associated with promoting tobacco products. Among men, the results show that they smoke for pleasure and to socialize, and consider that women smoke to imitate men and feel powerful. Regarding cessation, women are ambivalent about quitting smoking or not, and men mention not needing professional support. For organizational barriers, women mention the cost of treatment and men, the distance to clinics.

Conclusion. Smoking cessation interventions should be proposed from an approach that involves changes in social norms, seeking a more equitable relationship between men and women. Therefore, there must be broad engagement from different sectors and not just at the health sector level.

## Introduction

Tobacco use is the leading cause of preventable death worldwide (Ng et al., 2014; World Health Organization (WHO), 2015). Globally, 942 million men and 175 million women (aged 15 or older) are current smokers (Drope et al., 2018). Approximately, 75% of daily adult male smokers live in countries with a medium or high Human Development Index (HDI), and 50% of daily female smokers live in countries with a very high-HDI (Drope et al., 2018). While overall trends of tobacco use among men are declining, tobacco use continues to rise significantly among women in developing countries (Amos, Greaves, Nichter, & Bloch, 2012; World Health Organization (WHO), 2012, 2015). Moreover, there is evidence of an increase in the prevalence of tobacco use among young people, perhaps driven by the marketing strategies of the tobacco industry (Drope et al., 2018). This phenomenon is particularly true among women, regardless of the HDI country category.

Gender is a social construct, which refers to the conformation of behavior determined by sex at birth (Fenstermaker & West, 2002). The assigned gender role shapes the activities, expectations, and opportunities that are considered appropriate in a given socio-cultural context (Fenstermaker & West, 2002; Kågesten et al., 2016). The tobacco industry exploits gender norms to improve marketing goals. For example, smoking has been presented to women as feminine, attractive, and rebellious; whereas for men, it has been presented as strength, virility, independence, and mystery, which are characteristics of the ‘masculine ideal’ (Dutta & Boyd, 2007; Hafez & Ling, 2005). The tobacco industry also designs products directed at women. For example, long and ultra-long cigarettes have been developed to attract women by provoking feelings of independence, liberation, thinness, success, glamor, and taste (Greaves, 2015; Toll & Ling, 2005). Gender role differences can be seen throughout the spectrum of the tobacco use experience and include initiation, maintenance, cessation and relapse (Becker, McClellan, & Reed, 2017). It has been reported that women were less likely to quit smoking than men (Smith et al., 2015). However, there is limited literature on this topic in Latin America. In Argentina, women are less likely to quit when compared to men, which is an effect observed independently of age group (Niedzin et al., 2018).

### Study context

In 2003, Mexico signed the Framework Convention on Tobacco Control (FCTC) (Decreto Promulgatorio del Convenio Marco de la OMS para el Control del Tabaco, adoptado en Ginebra, Suiza, el veintiuno de mayo de dos mil tres, 2005; United Nations Treaty Collection, 2003) and, since then, has promoted tobacco control through key policies, which are specified in the General Law on Tobacco Control (GLTC) (Congreso de los Estados Unidos Mexicanos, 2018). These policies include the following: (a) partial prohibition of advertising, promotion and sponsorship (i.e. it is only allowed in magazines for the adult population) (Congreso de los Estados Unidos Mexicanos, 2018), (b) implementation of tobacco product taxes on packets of cigarettes and a recent adjustment for inflation accumulated in the period 2011–2019 (Congreso de los Estados Unidos Mexicanos, 2019; Waters, Saenz de Miera, Ross, & Shigematsu, 2010), (c) inclusion of pictograms and health warnings on the packs (i.e. 30% on the front, 100% on the back and one side of the box) (Salud pública y tabaquismo, volumen II, 2013), (d) prohibition of the marketing of nicotine-containing products (e.g. e-cigarette, IQS) (Decreto por el que se modifica la Tarifa de la Ley de los Impuestos Generales de Importación y de Exportación, (2020), (e) banning of the importation and commercialization of nicotine-containing products (Congreso de los Estados Unidos Mexicanos, 2018; Zavala-Arciniega, Gutiérrez-Torres, Paz-Ballesteros, Reynales-Shigematsu, & Fleischer, 2019), and (f) enaction of 12 smoke-free state laws to protect 50% of the Mexican population (GATS, 2017; Tobacco Free Kids, 2019).

Mexico is among the top five Latin American countries with the highest prevalence of tobacco consumption. With a population of 120 million, over 14.3 million adults (16.4% of the total adult population) are current smokers in Mexico (25.2% men, 8.2% women), with rates of tobacco use rising 5% among women and only 2% among men between 2009 and 2015 (Drope et al., 2018; GATS, 2017). According to the Global Adult Tobacco Survey (GATS), 80% of Mexican smokers planned to, or were thinking about, quitting, and 56.9% (57% men, 56.4% women) made a quit attempt in the past 12 months. 19.3% (21.8% men, 14.7% women) of Mexican smokers were advised to quit by their primary healthcare provider (GATS, 2017). No comparative study on cessation by gender has been conducted in Mexico. Although smoking prevalence and cigarette use per day are higher among men when compared to women, women still develop dependence faster after initial use, report shorter and less frequent abstinence periods, and smoke for longer periods in their lifetime (Becker et al., 2017; Smith et al., 2015). Additionally, women hold stronger outcome expectancies about using smoking for mood-management (i.e. decreased stress, nervousness, depression) and weight and appetite control (Greaves, 2015; Piñeiro et al., 2016; Westmaas & Langsam, 2005). These factors might be some of many that could explain the gender-related disparities in smoking cessation.

The objective of this study was to explore the gender-related beliefs and attitudes about tobacco use and smoking cessation in Mexico. Given the impact of tobacco use and the gender differences (in both tobacco consumption and smoking cessation), it is important to develop gender-adapted smoking cessation interventions in Mexico, and the Latin American, as part of our immediate global public health priorities (Greaves, 2015).

## Material and methods

This study was part of the ‘*Vive sin tabaco … ¡Decídete!* smoking cessation study’ – a collaboration between The National Institute of Public Health (INSP) in Mexico and The University of Kansas Medical Center (KUMC) (Cupertino, Cartujano-Barrera, Perales, et al., 2019; Ponciano-Rodríguez et al., 2018). The study was conducted at two primary healthcare clinics of the *Institute of Security and Social Services of State Workers* (*ISSSTE* acronym in Spanish), located in Mexico City. ISSSTE clinics serve insured employees of the state and provide health coverage to approximately 30 million Mexican citizens (INEGI, 2015). These healthcare clinics were selected because they have smoking cessation programs. This study was conducted between February and July of 2015. Study procedures were approved and monitored by INSP-Human Subjects Committee (CI: 1197).

The research team designed the study, carried out the fieldwork, analyzed the collected data and wrote this document. The team was made up of multidisciplinary professionals with several years of experience in the topic of smoking cessation and the use of various information-gathering techniques, such as semi-structured interviews and other qualitative techniques. Likewise, the team has participated in numerous research and intervention initiatives on the subject, mainly from the perspective of public health.

### Study design

Because of the complexity in understanding and approaching the beliefs and attitudes of human subjects, the team determined that the study was to be conducted using qualitative methods. This decision considered that qualitative research corresponds to the study of human processes in which the answers cannot be translated into numbers and revolves around the understanding of subjective experiences (Bosi, 2012). The research was developed based on grounded theory, which, according to Cuesta Benjumea (2006), consists of the identification of central and emerging categories or units, from which new relationships are established.

### Participants

After using *Vive sin tabaco … ¡Decídete!*, an eHealth decision-making smoking cessation intervention (Cupertino, Cartujano-Barrera, Perales, et al., 2019), all participants were directly invited be a part of the present study. The choice of using units of observation (e.g. human informants), allowed a deeper and more detailed approach to the beliefs and attitudes of women and men who smoke, as well as their relationship to gender. The study performed a purposive sampling of complete collection (Martínez-Salgado, 2012), focused on including the participants who meet the following specific criteria: (1) self-identified current smokers, (2) ≥18 years of age, (3) had an active telephone number to complete an interviewer-initiated follow-up phone call at 12 weeks, and (4) accepted to be interviewed personally at their primary health care unit.

### Procedure

Individuals who were eligible to participate in the study were scheduled for an in-person appointment with the research staff. In total, 21 people were contacted for an interview; however, five women and two men did not attend their scheduled interview appointments. Therefore, the final sample included 14 participants, of which eight were women and six men.

During the in-person appointment, staff discussed all aspects of study participation and confidentiality, answered any questions, and guided eligible smokers through the process of verbal consent. Participants completed a survey on age, sex, smoking patterns (daily or occasional smoking), years of smoking, and history of quit attempts. Semi-structured interviews followed a guide developed by the research team (in Spanish).

**Figure 1.**
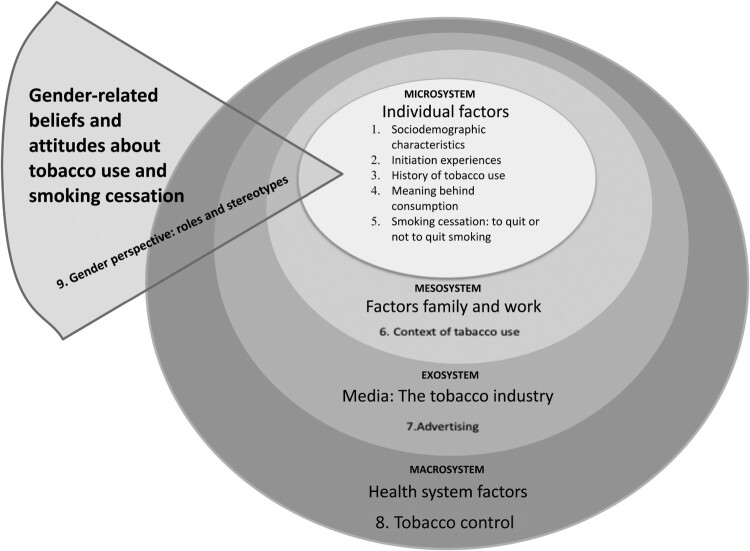
Topic and subtopic according an Ecological Model.

The guide explores eight dimensions: 1 sociodemographic data, 2 initiation experiences, 3 history of tobacco use, 4 meaning behind consumption, 5 smoking cessation: to quit or not to quit smoking, 6 context of tobacco use, 7 advertising, and 8 tobacco control. The guide considers the levels of the ecological model, which supports the analysis (Figure 1). With a cross-cutting focus on gender perspectives (i.e. roles and stereotypes), the interviews were conducted face to face, by a member of the research team, with experience in conducting interviews. The interviews lasted approximately 30 minutes: they were audio-recorded and occurred in a single encounter. Finally, 12 weeks after enrollment, an over-the-phone assessment was conducted to assess self-reported smoking abstinence (e.g. no cigarettes in the past 7 days) (Fiore et al., 2008).

### Analysis

All interviews were transcribed in a word processor and all identities replaced with a study ID to maintain confidentiality. From the eight dimensions established in the interview guide, a coding manual was (1) generated with operational definitions for each code and (2) created to categorize and retrieve quotes about each theme. The manual was later extended with emerging codes during the re-readings of the interviews. Two coders independently analyzed the data and identified codes within core domain categories with emerging themes. Inter-rater reliability was assessed for four groups of codewords (92% agreement).

The analysis used the inductive method, which is guided directly from the data collected, rather than from priori assumptions from other investigations (Amezcua & Gálvez Toro, 2002). The ecological model was considered because it accounts for the multidimensionality of our object of study and structures the results, based on several factors: (a) Individual factors – the experiences and meanings of smoking, ability to act, perceived social norms, perception of differences by gender, (b) Family and work factors – spaces that contribute to the construction and maintenance of gender roles, (c) Mass media- the tobacco industry and/or governments construct gender norms, and (d) Health system factors – the influence of the health system in the organization of tobacco cessation services. The research used Atlas-Ti® (v.7) to support the coding and analysis of the information collected from the semi-structured interviews.

### Results

#### Characteristics of the study participants

Women (*n *= 8) were between the ages of 19 and 63, with half being 35 years of age or younger. Seven women were daily smokers; four women had over 25 years of smoking history; six women had prior smoking quit attempts; and five women reported abstinence in the last 7 days at the 12 weeks follow-up. The six male participants were between the ages of 30 and 68 years old, with two-thirds being over 35. As in the case of women, most men (5 out of 6) were daily smokers, had smoked for more than 40 years, and had a history of quitting attempts. Only two of the men, however, reported abstinence at least in the last 7 days during follow-up (Table 1).

**Table 1.** Characteristics of the interviewees, Mexico City.

Participants	Age (years)^a^	Smoking patterns	Years of smoking^a^	Cessation attempts^b^	Number of cessation attempts^b^	Self-reported abstinence^c^
Women						
P1	19	Daily	7	Yes	2	Yes
P2	19	Non-daily	3	No	0	No
P3	26	Daily	10	No	0	Yes
P4	35	Daily	23	Yes	2	Yes
P5	57	Daily	43	Yes	8	Yes
P6	58	Daily	46	Yes	3	No
P7	58	Daily	44	Yes	4	No
P8	63	Daily	40	Yes	4	Yes
Men						
P1	30	Non-daily	18	Yes	1	No
P2	51	Daily	31	Yes	2	--
P3	63	Daily	52	Yes	10	Yes
P4	64	Daily	51	Yes	3	No
P5	68	Daily	50	No	0	Yes
P6	68	Daily	56	Yes	4	No

^a^ At the time of interview.

^b^ In the last 12 months.

^c^ No cigarettes (even a puff) in the past 7 days at 12 weeks of follow-up.

-- No data.

## Individual factors – the experience and meaning of smoking, ability to act, perceived social norms, and perception of differences by gender

### Experiences of different stages of tobacco use

Men and women participants reported that tobacco consumption is not constant over time because life events cause consumption to change. Imitation was the most important factor influencing smoking initiation. Men experienced their first cigarette in childhood, influenced by their family and as a way to bond with father figures. However, women reported smoking initiation as part of peer socialization during school years:

When I was little I got in between my dad's legs. He smoked cigars, ‘Faritos,’ and then he would let me try it. Well, I would ask and he would give. (M P3)

Outside of high school with the classmates, you see that they say ‘well it is just one cigarette, try it’. And that's where you start, debating whether or not to try it, a puff today, tomorrow another and that is how it happens. (W P7)

Men and women agreed that the increase in the frequency of consumption occurs when they achieved a certain degree of independence. Men recognized their increase in tobacco consumption to be associated with higher income, socialization, and pleasure:

[…] You smoke because you are in a good mood or because you are in a bad one, because you enjoy a good meal, lots of excuses … Talking about pleasurable things, sharing intimacy with your partner that also gives me pleasure and I do it. Well, everything can be a motive to smoke a cigarette. (M P2)

Women emphasized that they smoke cigarettes because they feel ‘supported’ and helps them ‘endure’ the responsibilities of modern life (i.e. being working women outside and inside of the home):

[…] I think it makes me feel like … protected … as if I can grab on to you (cigarette), because I do not have anyone else to hold on to apart from you (cigarette). That is, like you are my partner or my companion, it sounds silly, but it is like that. (W P5)

### About tobacco consumption and influence smoking maintenance

Results showed a difference between women and men regarding social norms. While men did not care about smoking in a public place, women who were over 50 years of age reported only smoking in places ‘allowed by social norms.’ The older women (e.g. aged 50 years or older) did not smoke while walking down the street and even criticized such behavior in other women. Interestingly, smoking in public areas was not a problem for younger women, nor for men.

[…] a man stands at the bus stop and there he is smoking. The woman represses herself a little more, well, at least I do … . (W P7)

[…] my wife does not like to smoke in the street, she smokes in the house, she obviously does not smoke in the office, and neither in the street. In fact, she does not carry cigarettes, she has her pack in the house. She only smokes in the house, or when we go out with a relative. (M P5)

Responses also reflected a person's ability to act with initiative, willpower, and external stimuli as reinforces to support the decision to quit smoking.

Well yes, my lack of willpower probably, my lack of willpower, because I do like to smoke, I feel good smoking, no? So to stop doing something that one likes is very difficult, right? (W P8)

Well, what could it be – surely not the psychological pressure. It's the lack of character, to say enough already, the temptation to have the cigar there. Well yes, I do not see any other reason. (M P5)

## Family and work factors – spaces that contribute to the construction and maintenance of the roles gender

Family ties and interactions with family members can produce both motivating elements and barriers to quit smoking for both genders. Quitting smoking can be more challenging when a person has family members who share in the activity of consuming tobacco with them. This is thought to be secondary to implied permissiveness and reinforced socializations. However, family can also represent factors that motivate the smoker to quit smoking. Some women, for example, reported having experienced being ‘caregivers’ of older family members and not wanting to place that burden on their own children. This then became a motivation to quit smoking and improve overall health. In other cases, quitting was seen as a means to maintain a romantic (and smoke-free) relationship.

My dad always smoked, and I think it did influence me. A type of social prototype that I watched smoking, something like that … . (M P2)

[…] I want to have a healthier lifestyle and I do not want to give my children … for example, I take care of my mother and she is older. So, I would not want for my children, and not because my mother gives me trouble as it is part of the activities one does, but I would like to give my children that type of trouble, if I get cancer, something like that … . (W P5)

[…] I do not know how to be an example for someone else, my nephews in this case … when I made the decision to not smoke was when my father smoked the most and I did not like it, so, well … I am going to smoke too. (M P1)

Although participants considered themselves respectful of workplace smoking restriction, this did not serve as an impediment to smoking during work hours, including in areas where it was prohibited. We identified gender-related differences in these behaviors.

Two of the women interviewed reported experiencing anxiety as a product of their responsibilities. These were, however, not only related to work but to the care of their children. This can be likened to having a double shift, which is attributed to females who are caregivers and workers. Another woman interviewed reports that she did not smoke during a vacation period because she was at home free of work pressures.

A retired woman explained that she had been used to working outside the home and that she does not like the activities associated with staying home. She states that lifestyle changes due to her retirement have been difficult, which is causing her states of anxiety and loneliness and increasing her tobacco use. She endorses that these emotions are linked with the maintenance and increase in tobacco consumption.

Multiple workloads are repeatedly reported in women's interviews, this aspect does not appear in men's narratives:

[…] it was only when I would go to work and in the high-pressure seasons, then a lot of pressure would cause me a lot of anxiety, the responsibility of my children, caring for them, even though others would take care of them and everything, but it always would cause it … . (W P5)

Young women report consuming more tobacco due to responsibilities related to being a student and fulfilling a job simultaneously. The ‘double shift’ analogy (e.g. having to work and be a student) of women appears to be a part of their lives from youth, or at least is more easily identified and related to tobacco consumption than in their male counterparts. Conversely, young men refer to using tobacco as part of socialization with their peers:

[…] I had to work, I came to the place of work. I went to my classroom and then I came home at night and had to do homework, so I started smoking a lot; before, occasionally a cigarette, then when I entered the classroom of two or three cigarettes. (W P2)

Well, yes, I smoked more frequently when I was in college or at parties too … . (M P1)

Despite being retired, some older men continue to work outside the home thus remaining in their role as providers:

[…] perhaps because of the decrease in my professional activity, it is as if one doesn't have much to do but to be forced to forget about the cigarette. Because when I am busy, pressured to hand over some work, I forget about the cigarette. But also sometimes with work's pressure and if I have cigarettes close by, when I least think about it, I’m already smoking. (M P6)

## Mass media: the tobacco industry or governments construct gender

Participants who talked about advertising and tobacco, remember few women smoking in ads or that it exacerbated masculine attributes. Among the most memorable messages for both men and women was part of a marketing campaign of one of Mexico's best-selling brands – an allusion to the figure of a cowboy.

[…] Marlboro I even remember the little music, the men there with their … in the mountain with their little lights, no? Or the very elegant guys with their cars smoking, no? Like it gave them a special presence, no? But men, they have never done publicity with women smoking, however, because of freedom we did it. (W P8)

The memory of the Mexican actresses was so relevant that some participants stated that they would think of them as a role model and/or a figure to admire during the act of smoking. The image Mexican actress transmitted was ‘elegant and strong,’ which appeals to a new way of being for women.

*Men – influence through the movies*

[…] it would show up in some part of cinema and things of that type and well, you obviously see it in the movies, not only men. In that time there were already women that smoked, generally very elegant women with their cigarette. Maybe that gets into you psychologically, and even if you say it's not true, that you didn't consider the models, well, I think that those type of images did influence. (M P5)

Men identified ‘advertising’ as a barrier to quitting smoking and ‘publicity’ as a positive reinforcement that reminds people of the association of cigarette consumption with pleasure.

[…] I have a bad habit, I think that is it, but this does help psychologically. It does help, in terms of the television, psychologically I notice that I am not thinking about cigarettes, but when I am watching a movie and they start smoking, I crave it. I think about cigarettes in the back of my head, but I do not go smoke, but it simply gives me an urge to smoke. (M P3)

One of the women interviewed recognized that advertising could indirectly promote smoking. She noted that women do not go smoking outside as men do in advertisements. Several women also identified that, in marketing, when a woman started smoking it was because they felt they acquired ‘values’ related to their own freedom/emancipation as women. Although overall there are currently many more restrictions on the promotion of tobacco products, the powerful symbolism represented in these commercials still persist.

We asked participants if they thought there was any difference in the reasons that men and women smoke. Women mentioned different points of view. One woman reported that they smoke according to their mood, while men smoke to pass time, feel pleasure, or feel masculine. Another woman said that ‘society’ can promote consumption. There were those who reported that there is no difference between men and women:

Well, I say we are the same, because there are those that smoke for pleasure, because of stress, and for any other concern, and men too, no? Regarding attempts … it's the same, no? There are determined men and determined women. (W P6)

A theme that emerged in the narratives was of smoking being linked to feelings of freedom (i.e. smoking as a symbol of liberty):

No, I think that now is a time of a lot of freedom, because I see young girls smoking in the street, they are talking and each has their cigarette as it is no longer frowned upon, no? (W P8)

However, it seems that for women, there could be a comingling of feelings of pleasure and guilt. Smoking destroys the ideal image of ‘being a good mother’ and good maternal role model. An existential question exists of, how can one be a good mother if, by their actions, they are setting a bad example for their children or family. So, although, as it would seem, women enjoy the pleasurable feelings of smoking, there is an underlying element of shame and guilt that is not seen in their male counterparts.

[…] not only does look bad, it does not give you a good image, it's like you’re a woman and you’re going to have children, you have to take care of it, how you are smoking? (W P2)

On the other hand, most men do not perceive differences either in the reasons for smoking or in success in quitting. However, they suggested believing that women who smoke are doing so in order to (1) imitate men, (2) feel powerful, or (3) demonstrate that they have the same rights as men.

… I do not know, (women) try to give a difference appearance, to imitate a man or give the appearance of a little man, as if that is going to give them power or … , that she is going to be more of a women. (M P4)

Along the same thematic narratives of exploring the relationship between the habit of smoking and (1) the search for equality in women, (2) the exercise of power, and (3) the exercise of the same rights, another idea, regarding female smoking behavior, was introduced as a ‘delicate’ way of smoking:

'They have a more delicate way of smoking than men but, overall I think it's the same. (M P3)

Just as women want to show that they have the same rights and capabilities as men, if men smoke, why not women? (M P1)

In addition, participants escribe some differences. When women are asked about gendered reasons for smoking and quitting, an important theme that was mentioned was feelings of increased masculinity in men, and perceptions of women as primary caregivers, etc. As mentioned above, one of them is considered to be related to masculinity:

I think men smoke for masculinity. The majority of the women that I know, it seems we smoke due to anxiety or loneliness – at least the ones that I know. (W P8)

This underscores the recurring idea of women's smoking behavior being tied with feelings of inadequacy and dependence to men. The suggestion is that for men, smoking is an ‘act of will,’ whereas for the woman, it is done in an effort to equate to men as the ‘perfect model.’

Another important thematic idea is the perception of women being in the role of caregiver, which is implied throughout the statements and reflections previously discussed. This further highlights the gender norms that facilitate smoking, as women are constantly perceiving their gender roles in the context of pleasing others.

An interesting aspect is that men did not have answers when asked the same type of questions, which suggests that men did not know why women start or stop smoking:

[…] I don't know what would be the motives for a woman to quit smoking. (M P1)

Young participants from both genders reported experiencing events that made them feel the need to relieve stress and/or escape, which caused their smoking to increase.

Well sometimes when I get very stressed, I really want to smoke, because for me it's like de-stressing, I feel more relaxed. (W P1)

Especially when I have stressful days I feel the craving to smoke, as if to vent. (M P1)

Although the majority of smokers reported wanting to quit smoking, they were aware of the presence of barriers, both at the individual and organizational level, that prevented them from achieving this goal.

Individual barriers include being ambivalent about quitting smoking or not. A comment that caught the attention of interviewers was expressed by one of the women. She said that she was not interested in quitting because ‘she was simply not motivated to smoke or not smoke.’ Additionally, one of the participating men (who had tried to quit before) said that he did not need support from a health professional.

 … no, I have not had any motivation to quit smoking and to continue smoking either, it's like … , if I want to smoke I smoke and if not, I mean here in the clinic I could take my gown off and go to the street, smoke a cigarette and come back with no problem, but I don't do it because I will smell, the cigarette smell can be smelt so I prefer to avoid it in that sense. (W P3)

mmm, no, in fact I tried to do it of my own will and decision, I do not smoke much, I say well I can stop smoking, it is just not buying anymore and that's it, saying I’m not going to smoke and I don't smoke. (M P1)

## The influence of the health system in the organization of tobacco cessation services

Organizational barriers referring to health services include hours of care, lack of treatment, costs, and care staff. Of those with a past history of attempting to quit smoking, both older men and women relied exclusively on ‘willpower’ without the support of a health professional. Overall, men sought more professional help and mentioned more references about the smoking cessation programs in the clinics when compared to women. One woman said that medication was a limitation to want to qui smoking. None of the women interviewed spoke of problems of weight gain or other potential negative effects as barriers to quitting smoking.

For some women, the cost of treatment was identified as a barrier to accessing smoking cessation services:

[…] we cannot be in a program because the medications are expensive and we prefer buying cigarettes to buying medicine but if we are supported, it would be too much to say ‘if they give it to me I’m not going to take it’, right? (W P7)

Men also reported that the way that these specialized clinics are organized, combined with the distance from their home, was a barrier to taking advantage of the cessation services and quitting smoking.

I don't come because of distance, good pretexts, right? Yes, I am very far from my house. (M P2)

Regarding organizational barriers, both women and men in the workforce mentioned that they were not able to participate in smoking cessation programs because their work, home responsibilities hindered them from attending sessions at the times they were available.

Well … , well I don't know, I work in the afternoon … but I don't know neither the day nor times they have them. (W P3)

‘[…] I had come to the program they had here in the clinic but I got unmotivated a little because they said that had to form the group, because they were finishing up, something like that, that I had to wait for them to start again. (M P1)

## Discussion

In light of this study focusing on the gender perspectives that facilitate or prevent tobacco use, the results demonstrate various overarching themes of discussion tobacco-related gender differences: tobacco initiation, event affect smokers, reason to quit smoking, the media power of the tobacco industry, and personal and organizational barriers to smoking cessation. An underlying condition that many women expressed throughout the narratives is feelings of depression and anxiety, which were attributed to the multitude of gender-related roles women fulfill. These results are supported in other studies (Etter, Prokhorov, & Perneger, 2002; Samet & Yoon, 2010), and according to the National Survey on the Use of Time 2014, women dedicate 28.2 hours per week to care activities (i.e. they take care of their home, dependents, and relatives), while men dedicate 12.4 hours per week for the same tasks (INEGI-INMUJERES, 2015).

During the narratives, an analogy of women having a ‘double-shift’ of having to work inside and outside of the home, whereas men typically do not, further illustrate the inequalities of work responsibilities. These pressures on women are explained to favor continuity of tobacco consumption. These pressures also inherently predispose people to acquiring mental disorders. A balance should be proposed in the allocation of tasks (mainly domestic tasks), so that both women and men have a better quality of life. Future studies should assess depression and anxiety by gender as moderators of smoking cessation.

Asking the question, what is your main reason to quit smoking? The responses of the participants reflect the general attitudes and perception of traditional gender roles in Mexico, where women are the primary caregivers and rarely receive care, and men are the societal role models. For the women interviewed in this study, their health and reluctance to be a burden on the family were the main reasons for quitting; while for the men, their family and ‘not being a bad example’ were the main reasons. These findings suggest that future smoking cessation interventions should target these reasons to quit smoking and include them in the cessation plan. The social interaction of people and their contexts (i.e. family, work) make the cessation process complex (Solano, García-Tenorio, & De Granda, 2003). It is not only an individual action to quit smoking. Public health researchers have to implement actions and changes at the level of both social and cultural structures (institutions), so that these actions and changes can reach people (Castiel & Álvarez-Dardet, 2010). For example, educational campaigns that promote the agency of women in health issues.

The results of this study highlight differences between women and men, with regards to tobacco use initiation. For males, tobacco initiation within the family and starting to smoke at a younger age shows ‘the reproduction of the values of masculinity’. For women, tobacco use initiation was with friendships, at school, and out of curiosity. Women started to use tobacco at an older age compared to men. These results are similar to the ones reported by Ponciano-Rodríguez (2001), where she explains that tobacco use initiation is influenced by friendships, although without difference between men and women.

Various life events can affect smokers’ quantity and frequency of tobacco use. Women report that work and family-related stressors can affect their desires for smoking and lead to feelings of loneliness; whereas for men, having economic capacity and independence are the main contributing factors. For both, advertising represented a factor that increases consumption. In a paper written by Kaufman and Nichter (2001) that analyzes the marketing of tobacco to women, the authors reflect that in the same way tobacco industries have exploited gender stereotypes to increase tobacco consumption among susceptible populations, public health should incorporate gender perspective to promote health behaviors that contribute to gender equality.

Social interactions are full of emotions and affect (Collins, 2009). For women, consuming tobacco becomes a necessity since it provides them with ‘company,’ and they make reference to the cigar as if it is ‘another being.’ Therefore, quitting smoking means breaking a relationship, which may be one of affection, trust and fidelity (Ponciano-Rodríguez & Morales-Ruiz, 2008). It has been reported that Mexican women are more likely to quit smoking when they go to support groups since they see it as a form of socialization, which can help avoid depressive states (Ponciano-Rodríguez & Morales-Ruiz, 2008). At the smoking cessation support clinics, women give each other feedback and talk about their health status and life events that may cause stress-related increases of tobacco consumption. These groups should be a support of the situations that modern daily life imposes on women.

For women, smoking is associated with guilt and fear, due to the negative perception of being the caregiver and providing a bad example for the family and children in the home. Young women see it as an escape or relief and do not identify the consequences or damage it causes to their health (Nerín & Jané, 2007).

Incorporating these emotions in the process of quitting smoking should be beneficial in tobacco control policies. However, there must be clarity in the way these strategies are written, since they must encourage ‘empowerment’ behavior towards women. Women must feel emboldened to take control of their lives and bodies, so they can understand the meaning that smoking has to them. It is convenient to avoid actions that provoke more feelings of guilt or an excess of responsibilities that add to those that historically are assigned to women.

For men, the relationship to the cigarette is less affective. Cigarette consumption is a behavior that can be modified, not without being aware of how complex it is to stop smoking. Unlike women, who tend to view cigarettes as ‘company’ or an actual entity, men perceived cigarettes as a means for achieving pleasure, particularly sexual pleasure -as social facilitator. This behavior has been influenced by the mass media, as advertising has romanticized the act of smoking, which inherently makes it more attractive.

A similarity found between young men and women, is that both smoke for relief. This may mean that there is already an awareness of gender equality (i.e. smoking for freedom and relief to reduce the stress produced by the modern world); however, it would be premature to consider this a as conclusive finding. This only motivates the interest to deepen the study on consumption habits among young people.

As referred to by Collins (2009), the symbols of group belonging provoke emotional and affective energy, their main source being the media. The institutions are producers of the social coercion necessary for the functioning of society, mainly government and religion as an ideology of moral issues. These release devices (through advertising) that function as mechanisms that influence the behavior of people and their interactions with the social environment where they were born and developed, which assigns them a position within the social structure and determine their way of life (Commission on Social Determinants of Health, 2008).

In that sense, tobacco advertising was key to the initiation of smoking. Even though there had been no television and radio advertising for tobacco products in Mexico for over ten years, most of the individuals remembered the majority of ads, which is a consequence of the ‘mediatic’ power that the tobacco industry has had in society. The media determines the elements of relevance for producing and reproducing the symbols that influence smoking and quitting rates. This has been one of the main pillars of the promotion and sale of cigarettes made by the tobacco industry. The media's power of the tobacco industry has an influence on culture (Chomsky & Herman, 1990). Through the power of advertisement, specifically via social media, people are able to escape the pressures of their everyday life and broaden the reach of their influences. Social media allows for the transmission of the symbols of smoking, while creating a ‘sphere of reality’ that is able to be shared by all (Schutz & Luckmann, 1989).

The media has had a significant influence on tobacco consumption and on what it means to be a man or a woman. Most of the people interviewed lived their adolescence during the 1960s and 1970s, and it is during this period that national and international social movements of transcendence was started. At this juncture point in time, one can see a metaphorical ‘before and after’ effect of the social behavior of people, particularly among women during the feminist and student movements. In these years, smoking was ‘normalized’ and made public for the masses to partake in. Smoking was common in men, schools, workplaces and in all public spaces, and in some cases, smoking was even stimulated. Also, during this period was the rise of Mexican cinema as a main component of cultural reproduction. The Mexican writer Carlos Monsiváis, said that ‘cinema was and is an excellence the space of the masses, the film industry and it is important to recreate, reflect, flatter or criticize with discretion its public and the culture of where it comes from and that enriches, modifies and refines’ (Monsivaís, 2013, p. 173). This medium is key for the reproduction of images that provoke and incite tobacco consumption and also for the propagation of behaviors and roles, according to sex. As Monsiváis described, cinema transfers a ‘sensitivity translated into images,’ when it refers to the construction of ‘feminine sensitivity.’ It has been shown that the beginning of tobacco consumption is influenced by film and television (World Health Organization, 2016).

Although there has been no advertising in the media in Mexico since 2004, the population of participants in this study still remembers advertisements of recognized brands that were broadcasted more than 15 years ago. This highlights the relevance of stricter tobacco control policies that are needed, not only at the local level, but also at the international level.

Studies of the report of gender difference in tobacco cessation, suggesting that there are social/cultural factors and changes in the context that play an important role in developing gender differences in tobacco cessation (Jimenez-Rodrigo, 2015; Smith et al., 2015). In our results, men and women reported that their main reasons for quitting smoking are to maintain ‘health and family.’ Throughout the interviews, participants highlighted the ritualistic nature of smoking that leads to mindless usage. They also noted that, although there may be desires for quitting smoking, physical dependence (i.e. nicotine addiction) may be a hinderance to the ‘action’ of quitting. Among women, the impediment to develop their daily activities in their various roles is a motivation to quit smoking.

The women stated that they have individual reasons that prevent them from being able to start the process to quit smoking. These are referred to as ‘family problems,’ which the participants refer to as ‘pretexts.’ While men mention that they find it harder to stop smoking because tobacco promotes socialization. Among the barriers that prevent smoking cessation, women identified the ‘cost of treatment,’ while men identified the ‘challenge to cease socialization’ as main barriers. In other words, the feminine construct in Mexico implies that women put family problems, among other reasons, before their care; while men struggle with social aspects of masculinity in Mexico. The dominant male role, as a provider, seems to predominate until now as the men in our study did not identify the cost of a smoking cessation service as a barrier.

A limitation of this study is generalizability. Our study participants were smokers with state health coverage in Mexico, which may have led to an increase of access to health services than the majority of the Mexican population. Likewise, our participants were employees or family members of state employees, which implies likelihood of increased financial means compared to other Mexican people. Although the nature of qualitative research does not allow nomothetic generalization (Martínez-Salgado, 2012), our study presents findings that allow a greater understanding of the complexity of gender constructions in Mexico and their relationship with tobacco consumption. Therefore, results can be transferable to very specific contexts, where the intention is to deepen the knowledge in the same way that we did here.

To the best of our knowledge, this is the first study to explore tobacco-related gender differences among Mexican smokers. In this study, gender differences were identified in smoking initiation, tobacco use, and in the perceived barriers and facilitators for cessation among Mexican smokers. Future smoking cessation research or interventions can build upon these qualitative findings to develop and implement gender-appropriate tobacco control efforts in Mexico.

Despite participants’ knowledge of the health consequences caused by tobacco, and living through a tobacco-related familial death, some participants interviewed at the time of the follow-up had not been able to quit smoking. This demonstrates the great complexity of the problem of smoking and the importance of undertaking novel strategies for cessation in Mexico. Mobile health interventions are a promising mode to enhance smoking cessation treatment in Mexico (Cartujano-Barrera et al., 2020; Cupertino, Cartujano-Barrera, Perales, et al., 2019; Cupertino, Cartujano-Barrera, Ramírez-Mantilla, et al., 2019; Ponciano-Rodríguez et al., 2018). Developments in the sophistication of mobile technologies allow for flexible delivery of text messages, with algorithms used to tailor content of motivational and behavioral needs for smoking cessation (Kong, Ells, Camenga, & Krishnan-Sarin, 2014).

## Conclusion

To achieve gender equality in the access to health treatment and care is one of the objectives of public health. In this study, we analyzed the perceived gender roles between self-identified men and women, and looked at how these gender norms shaped their introduction to smoking and the barriers for cessation. The stressors of gender inequalities in the Mexican society should not be overlooked. Interventions to quit smoking should be proposed from an approach that involves changes in social norms by seeking a more equitable relationship between men and women. For women, cessation interventions should focus on the theme of empowerment, by ensuring that they feel validated, included, and visible, especially given the context of living in a patriarchal society. General masculine qualities discussed throughout the narratives were characteristics of responsibility, strength, and protective attitude. These characteristics can be used positively to encourage social chances and to support families and communities. Therefore, there needs to be a broad commitment to addressing these issues.

## References

[CIT0001] Amezcua, M., & Gálvez Toro, A. (2002). Different Approaches to Qualitative Health Research Analysis: A Critical and Reflective View [Los modos de análisis en investigación cualitativa en salud: perspectiva crítica y reflexiones en voz alta]. *Revista Española de Salud Pública*, *76*(5), 423–436. http://scielo.isciii.es/scielo.php?script=sci_arttext&pid=S1135-57272002000500005&lng=es&tlng=es 12422418

[CIT0002] Amos, A., Greaves, L., Nichter, M., & Bloch, M. (2012). Women and tobacco: A call for including gender in tobacco control research, policy and practice. *Tobacco Control*, *21*, 236–243. 22166266 10.1136/tobaccocontrol-2011-050280

[CIT0003] Becker, J. B., McClellan, M. L., & Reed, B. G. (2017). Sex differences, gender and addiction. *Journal of Neuroscience Research*, *95*(1-2), 136–147. 27870394 10.1002/jnr.23963 PMC5120656

[CIT0004] Bosi, M. L. M. (2012). Qualitative research in collective health: overview and challenges. [Pesquisa qualitativa em saúde coletiva: panorama e desafios]. *Ciência & Saúde Coletiva*, *17*(3), 575–586. doi:10.1590/S1413-81232012000300002 22450397

[CIT0005] Cartujano-Barrera, F., Rodríguez-Bolaños, R., Arana-Chicas, E., Gallegos-Carrillo, K., Flores, Y. N., Pérez-Rubio, G., … Cupertino, A. P. (2020). Enhancing nicotine replacement therapy usage andadherence through a mobile intervention: Secondarydata analysis of a single-arm feasibility study in Mexico. *Tobacco Induced Diseases*, *18*, 36. doi:10.18332/tid/120076 32395099 PMC7206510

[CIT0006] Castiel, L. D., & Álvarez-Dardet, D. C. (2010). *La salud persecutoria: los límites de la responsabilidad*. Argentina: Buenos Aires.

[CIT0007] Chomsky, N., & Herman, E. (1990). *Los guardianes de la libertad. Traducción de Carme Castells*. Barcelona, España: Grijalbo Mondadori.

[CIT0008] Collins, R. (2009). Interaction Ritual Chains *[Cadenas de rituales de interacción]*. Traducción de Juan Manuel Iranzo. Barcelona, España: Anthropos.

[CIT0009] Commission on Social Determinants of Health. (2008). Closing the gap in a generation: health equity through action on the social determinants of health: final report:executive summary. Geneva, Switzerland: World Health Organization. Retrieved July 15, 2020, from https://apps.who.int/iris/handle/10665/69830?locale-attribute=en&

[CIT0010] Congreso de los Estados Unidos Mexicanos. (2018). *Ley general para el control del tabaco. Diario Oficial De La Federación.* Retrieved July 15, 2020, from http://www.diputados.gob.mx/LeyesBiblio/pdf/LGCT_150618.pdf

[CIT0011] Congreso de los Estados Unidos Mexicanos. (2019). *Ley del Impuesto Especial Sobre Producción y Servicios. Nueva Ley publicada en el Diario Oficial de la Federación el 30 de diciembre de 1980. Última reforma publicada en el Diario Oficial de la Federación*. México: Diario Oficial de la Federación. Retrieved July 15, 2020, from http://www.diputados.gob.mx/LeyesBiblio/pdf/78_241219.pdf

[CIT0012] Cuesta Benjumea, C. (2006). La teoría fundamentada como herramienta de análisis. *Cultura de los cuidados*, 136–140. Retrieved February 15, 2021, from https://rua.ua.es/dspace/bitstream/10045/876/1/culturacuidados_20_19.pdf

[CIT0013] Cupertino, A. P., Cartujano-Barrera, F., Perales, J., Formagini, T., Rodríguez-Bolaños, R., Ellerbeck, E., … Reynales-Shigematsu, L. M. (2019). *“Vive Sin Tabaco … ¡Decídete!”* Feasibility and acceptability of an e-Health smoking cessation informed decision-making tool integrated in primary healthcare in Mexico. *Telemedicine and e-Health*, *25*(5), 425–431. 30048208 10.1089/tmj.2017.0299 PMC6916521

[CIT0014] Cupertino, A. P., Cartujano-Barrera, F., Ramírez-Mantilla, M., Rodríguez-Bolaños, R., Thrasher, J. F., Pérez-Rubio, G., … Reynales-Shigematsu, L. M. (2019). A mobile smoking cessation intervention for Mexico (Vive sin Tabaco … ¡Decídete!): Single-arm pilot study. *JMIR MHealth and UHealth*, *7*(4), e12482. doi:10.2196/12482 31021326 PMC6658244

[CIT0015] Diario Oficial de la Federación. (2020). Decreto por el que se modifica la tarifa de la Ley de los Impuestos Generales de Importación y Exportación. Retrieved July 15, 2020, from http://www.dof.gob.mx/nota_detalle.php?codigo=5586899&fecha=19/02/2020.

[CIT0016] Diario Oficial de la Federación. (2005). Decreto Promulgatorio del Convenio Marco de la OMS para el control del tabaco. D.O.F. 25-II-2005. Retrieved July 15, 2020, from http://www.diputados.gob.mx/LeyesBiblio/compila/regla/n144.doc

[CIT0017] Drope, J., Schluger, N., Cahn, Z., Drope, J., Hamill, S., Islami, F., … Stoklosa, M. (2018). *The tobacco atlas* (6th ed.). Atlanta, GA: American Cancer Society and Vital Strategies. Retrieved July 15, 2020, from https://tobaccoatlas.org/wp-content/uploads/2018/03/TobaccoAtlas_6thEdition_LoRes_Rev0318.pdf

[CIT0018] Dutta, M. J., & Boyd, J. (2007). Turning “smoking man” images around: Portrayals of smoking in men’s magazines as a blueprint for smoking cessation campaigns. *Health Communication*, *22*(3), 253–263. 17967147 10.1080/10410230701626901

[CIT0019] Etter, J. F., Prokhorov, A., & Perneger, T. (2002). Gender differences in the psychological determinants of cigarette smoking. *Addiction*, *97*(6), 733–743. 12084143 10.1046/j.1360-0443.2002.00135.x

[CIT0020] Fenstermaker, S., & West, C. (2002). *Doing gender, doing difference: Inequality, power, and institutional change* (1st ed.). New York, NY: Routledge.

[CIT0021] Fiore, M. C., Jaén, C. R., Baker, T. B., Bailey, W. C., Benowitz, N., Curry, S. J., et al. (2008). *Treating tobacco use and dependence: 2008 update*. Clinical Practice Guideline. Rockville, MD: U.S. Department of Health and Human Services. Public Health Service.

[CIT0022] Greaves, L. (2015). The meanings of smoking to women and their implications for cessation. *International Journal of Environmental Research and Public Health*, *12*, 1449–1465. doi:10.3390/ijerph120201449 25633033 PMC4344676

[CIT0023] Hafez, N., & Ling, P. M. (2005). How Philip Morris built Marlboro into a global brand for young adults: Implications for international tobacco control. *Tobacco Control*, *14*(4), 262–271. 16046690 10.1136/tc.2005.011189 PMC1748078

[CIT0024] INEGI. (2015). Health and social security *[Salud y seguridad social*.] Mexico: Author. Retrieved July 15, 2020, from https://www.inegi.org.mx/temas/derechohabiencia/

[CIT0025] INEGI-INMUJERES. (2015). National Survey on Time Use 2014 *[Encuesta Nacional sobre Uso del Tiempo 2014*]. Aguascalientes, Mexico: INEGI. Retrieved July 15, 2020, from https://www.inegi.org.mx/contenidos/saladeprensa/boletines/2015/especiales/especiales2015_07_2.pdf

[CIT0026] Jimenez-Rodrigo, M. L. (2015). Health policies from a gender perspective: An approach from the exam of tobacco control plans [*Las políticas de salud vistas desde el género: Una aproximación a partir del examen de los planes de control del tabaquismo*]. *Investigaciones Feministas*, *5*, 289–316.

[CIT0027] Kågesten, A., Gibbs, S., Blum, R. W., Moreau, C., Chandra-Mouli, V., Herbert, A., & Amln, A. (2016). Understanding factors that shape gender attitudes in early adolescence globally: A mixed-methods systematic review. *PLoS ONE*, *11*(6), e0157805. 27341206 10.1371/journal.pone.0157805 PMC4920358

[CIT0028] Kaufman, N. J., & Nichter, M. (2001). The marketing of tobacco to women: Global perspectives. In J. M. Samet & S.-Y. Yoon (Eds.), *Women and the tobacco epidemic: Challenges for the 21st century*. World Health Organization: Geneva, Switzerland, pp. 105-136.

[CIT0029] Kong, G., Ells, D. M., Camenga, D. R., & Krishnan-Sarin, S. (2014). Text messaging-based smoking cessation intervention: A narrative review. *Addictive Behaviors*, *39*(5), 907–917. doi:10.1016/j.addbeh.2013.11.024 24462528 PMC3980005

[CIT0030] Martínez-Salgado, C. (2012). Sampling in qualitative research. Basic principles and some controversies [*El muestreo en investigación cualitativa: Principios básicos y algunas controversias*]. *Ciência & Saúde Coletiva*, *17*(3), 613–619. doi:10.1590/S1413-81232012000300006 22450401

[CIT0031] Monsivaís, C. (2013). *Misógino feminista. Selección y prólogo de Marta Lamas*. México: Debate feminista: Editorial Océano, D.F.

[CIT0032] Nerín, I., & Jané, M. (2007). *Libro blanco sobre mujeres y tabaco. Abordaje con una perspectiva de género*. Zaragoza, España: Comité Nacional para la Prevención del Tabaquismo. Ministerio de Sanidad y Consumo.

[CIT0033] Ng, M., Freeman, M. K., Fleming, T. D., Robinson, M., Dwyer-Lindgren, L., Thomson, B., … Lopez, A. D. (2014). Smoking prevalence and cigarette consumption in 187 countries 1980–2012. *JAMA*, *311*(2), 183–192. 24399557 10.1001/jama.2013.284692

[CIT0034] Niedzin, M., Gaszyńska, E., Krakowiak, J., Saran, T., Szatko, F., & Kaleta, D. (2018). Gender, age, social disadvantage and quitting smoking in Argentina and Uruguay. *Annals of Agricultural and Environmental Medicine*, *25*(1), 100–107. 29575866 10.5604/12321966.1227646

[CIT0035] Pan American Health Organization (PAHO). (2017). Global Adults Tobacco Survey, Mexico 2015 *[Encuesta Global de Tabaquismo en Adultos, México 2015*]. Cuernavaca, México: INSP/OPS. Retrieved July 15, 2020, from http://www.who.int/tobacco/surveillance/survey/gats/mex-report-2015-spanish.pdf

[CIT0036] Piñeiro, B., Correa, J. B., Simmons, V. N., Harrell, P. T., Menzie, N. S., Unrod, M., … Brandona, T. H. (2016). Gender differences in use and expectancies of e-cigarettes: Online survey results. *Addictive Behaviors*, *52*, 91–97. 26406973 10.1016/j.addbeh.2015.09.006 PMC4644488

[CIT0037] Ponciano-Rodríguez, G., & Morales-Ruiz, A. (2001). *El Consumo de Tabaco en las Mujeres ¿Pose o Adicción? Parte I*. *Gac Fac Med*, 8–9.

[CIT0038] Ponciano-Rodríguez, G., & Morales-Ruiz, A. (2008). *La última bocanada: cartas de despedida al cigarro. Ortega y Ortiz Editores, S.A de C.V. Consejo Mexicano contra el Tabaquismo (COMCT). D.F., México.*

[CIT0039] Ponciano-Rodríguez, G., Reynales-Shigematsu, L. M., Rodríguez-Bolaños, R., Pruñosa-Santana, J., Cartujano-Barrera, F., & Cupertino, A. P. (2018). Enhancing smoking cessation in Mexico using an e-Health tool in primary healthcare. *Salud Pública de México*, *60*(5), 549–558. 30550116 10.21149/9348

[CIT0040] Samet, J., & Yoon, Y. (Eds.). (2010). *Gender, women and the tobacco epidemic*. Geneva, Switzerland: World Health Organization.

[CIT0041] Schutz, A., & Luckmann, T. (1989). *The structures of the life-world* (vol. 2, pp. 159–324). Evanston, IL: Northwestern University Press.

[CIT0042] Smith, P. H., Kasza, K. A., Hyland, A., Fong, G. T., Borland, R., Brady, K., … McKee, S. A. (2015). Gender differences in medication use and cigarette smoking cessation: Results from the International Tobacco Control Four Country Survey. *Nicotine & Tobacco Research*, *17*(4), 463–472. 25762757 10.1093/ntr/ntu212 PMC4402353

[CIT0043] Solano, S., García-Tenorio, A., & De Granda, J. I. (2003). *Iniciación y mantenimiento del hábito tabáquico. El paciente que va a dejar de fumar*. In M. Barrueco, M. Hernández Mezquita, & M. Torrecilla (Eds.), *Manual de prevención y tratamiento del tabaquismo* (pp. 107–140). ERGON, Madrid, España.

[CIT0044] Thrasher, F. J., Reynales-Shigematsu, L. M., Lazcano-Ponce, E., & Hernández-Ávila, M. (2013). *Salud Pública y Tabaquismo, volumen II. Advertencias sanitarias en América Latina y El Caribe. Instituto Nacional de Salud Pública*. Retrieved July 15, 2020, from https://www.insp.mx/images/stories/Produccion/pdf/130226_reporteTabacoVol2. Pdf

[CIT0045] Tobacco Free Kids. (2019). *El 50% de la población de México ya está protegida de la exposición al humo de tabaco de segunda mano, 2019*. Retrieved July 15, 2020, from https://www.tobaccofreekids.org/es/comunicados-prensa/2019_06_26_mexico

[CIT0046] Toll, B. A., & Ling, P. M. (2005). The virginia slims identity crisis: An inside look at tobacco industry marketing to women. *Tobacco Control*, *14*, 172–180. 15923467 10.1136/tc.2004.008953 PMC1748044

[CIT0047] United Nations Treaty Collection. (2003). WHO framework convention on tobacco control. Ginevra: OMS. Retrieved July 15, 2020, from https://treaties.un.org/Pages/ViewDetails.aspx?src=TREATY&mtdsg_no=IX4&chapter=9&clang=_en

[CIT0048] Waters, H., Saenz de Miera, B., Ross, H., & Reynales-Shigematsu, L. M. (2010). *The economics of tobacco and tobacco taxation in Mexico*. Paris, France: International Union Against Tuberculosis and Lung Disease. Retrieved July 15, 2020, from https://untobaccocontrol.org/kh/taxation/wp-content/uploads/sites/3/2020/01/Mexico-Report_April2010_Online-Final.pdf

[CIT0049] Westmaas, J. L., & Langsam, K. (2005). Unaided smoking cessation and predictors of failure to quit in a community sample: Effects of gender. *Addictive Behaviors*, *30*, 1405–1424. 15896921 10.1016/j.addbeh.2005.03.001

[CIT0050] World Health Organization. (2016). *Smoke-free movies: From evidence to action* (3th ed.). Geneva, Switzerland. Retrieved July 15, 2020, from https://apps.who.int/iris/bitstream/handle/10665/190165/9789241509596_eng.pdf?sequence=1

[CIT0051] World Health Organization (WHO). (2012). *Global report: Mortality attributable to tobacco*. Geneva, Switzerland: Author. Retrieved July 15, 2020, from http://www.who.int/tobacco/publications/surveillance/rep_mortality_attributable/en/index.html

[CIT0052] World Health Organization (WHO). (2015). *Global report on trends in prevalence of tobacco smoking*. Geneva, Switzerland: Author. Retrieved July 15, 2020, from http://apps.who.int/iris/bitstream/handle/10665/156262/9789241564922_eng.pdf;jsessionid=C03F0C7221513E8972C156E00BAD7194?sequence=1

[CIT0053] Zavala-Arciniega, L., Gutiérrez-Torres, D. S., Paz-Ballesteros, W. C., Reynales-Shigematsu, L. M., & Fleischer, N. L. (2019). Correlates of secondhand smoke exposure in public and private settings in Mexico [*Factores asociados con la exposición al humo de tabaco de segunda mano en lugares públicos y privados en México*]. Encodat 2016. *Salud Pública de México*, *61*, 591. 31314210 10.21149/9877

